# Altered multisensory temporal integration in obesity

**DOI:** 10.1038/srep28382

**Published:** 2016-06-21

**Authors:** Federica Scarpina, Daniele Migliorati, Paolo Marzullo, Alessandro Mauro, Massimo Scacchi, Marcello Costantini

**Affiliations:** 1 “Rita Levi Montalcini” Department of Neuroscience, University of Turin, Turin, Italy; 2 I.R.C.C.S. Istituto Auxologico Italiano Ospedale San Giuseppe, Piancavallo, Italy; 3 Department of Neuroscience, Imaging and Clinical Science, University G. d’Annunzio, Chieti, Italy; 4 Department of Translational Medicine, Università del Piemonte Orientale ‘A. Avogadro’, Novara, Italy; 5 Department of Clinical Sciences and Community Health, University of Milan, Milan, Italy; 6 Centre for Brain Science, Department of Psychology, University of Essex, UK

## Abstract

Eating is a multisensory behavior. The act of placing food in the mouth provides us with a variety of sensory information, including gustatory, olfactory, somatosensory, visual, and auditory. Evidence suggests altered eating behavior in obesity. Nonetheless, multisensory integration in obesity has been scantily investigated so far. Starting from this gap in the literature, we seek to provide the first comprehensive investigation of multisensory integration in obesity. Twenty male obese participants and twenty male healthy-weight participants took part in the study aimed at describing the multisensory temporal binding window (TBW). The TBW is defined as the range of stimulus onset asynchrony in which multiple sensory inputs have a high probability of being integrated. To investigate possible multisensory temporal processing deficits in obesity, we investigated performance in two multisensory audiovisual temporal tasks, namely simultaneity judgment and temporal order judgment. Results showed a wider TBW in obese participants as compared to healthy-weight controls. This holds true for both the simultaneity judgment and the temporal order judgment tasks. An explanatory hypothesis would regard the effect of metabolic alterations and low-grade inflammatory state, clinically observed in obesity, on the temporal organization of brain ongoing activity, which one of the neural mechanisms enabling multisensory integration.

When we eat, we are bombarded by multisensory information, including visual, auditory, tactile, gustatory and olfactory^1^ which need to be effectively integrated by our brain. Such multisensory integration deeply impacts on our appreciation of food^2^,^3^. Multisensory integration refers to the brain mechanism by which when two or more sensory stimuli occur at the same time and place^4^,^5^, they are typically bounded into a single percept, and detected more accurately than either stimuli alone^6^. Recently, there has been increasing interest in studying multisensory integration, and under what circumstances this process takes place. This is mostly due to the large impact multisensory integration has on how we perceive stimuli in the environment, including food^2^. For instance, Zampini and Spence^7^ investigated whether the perception of the crispness and staleness of potato chips was affected by the sound produced during the biting action. Results showed that varying the loudness or the frequency composition of the auditory feedback elicited during the biting action, altered the perception of both the crispness and staleness^8^. Similarly, Slocombe and colleagues^9^ investigated whether the perception of taste components within flavour could be altered by multisensory manipulations in texture. They asked participants to rate the sourness, sweetness and bitterness of a solid food substance, which was manipulated in texture. Results showed that a rough-textured foodstuff was rated as sourer than an otherwise identical smooth-textured foodstuff. What is more, eating does not only involve the integration of exteroceptive information, but also information from within the body, i.e. interoception. Interoception, defined as the sense of the physiological condition of the body, is a ubiquitous information channel used to represent the internal state of our body. For instance, fullness sensation in the stomach prompts the act of stop eating.

Obese patients have often disturbed eating behaviours^10^,^11^,^12^, considered as non-adjustable primary factor to being overweight^13^. Differences in food intake are influenced by individual characteristics that could magnify or minimize the genetic, physiological, environmental, psychosocial, cultural and cognitive risks^10^,^14^,^15^,^16^ for obesity.

Given the role of multisensory integration in eating behavior, and given the alterations of eating behavior in obesity^10^,^11^,^12^, we seek to provide the first comprehensive investigation of multisensory integration in obesity. Indeed only recently preliminary results were reported by Wan and colleagues^17^. They showed that obese people tend to respond more slowly to multisensory stimulation in an audiotactile discrimination task than underweight people.

Several factors impact multisensory integration. One such factor is the temporal relationships of the paired stimuli^4^,^18^. Accordingly, the more temporally coincident the stimuli, the larger the multisensory integration generated by their pairing^18^. While temporal synchrony generally results in a high degree of multisensory integration, paired stimuli do not need to be precisely synchronous in order to generate significant neural and perceptual changes. Indeed, multisensory integration occurs over a temporal interval called multisensory temporal binding window^19^ (TBW), or temporal window of integration^20^. TBW is defined as the range of stimulus-onset asynchronies (SOAs) in which multiple sensory inputs have a high probability of being integrated and altering responses^21^. TBW represents a measure of the effectiveness of multisensory integration mechanisms^21^,^22^. Previous studies have indeed shown that alterations of the TBW are strongly related to high-level cognitive impairments, including language and communications^19^,^22^.

Several experimental paradigms have been developed to assess the TBW. Two such experimental paradigms are the simultaneity judgment task (SJ) and the temporal order judgment task (TOJ)^21^,^23^. In the SJ, pairs of multisensory stimuli (e.g. auditory and visual) are presented at different SOAs and participants are required to judge whether the stimuli were presented simultaneously or successively^21^,^23^. In the TOJ, participants are presented with a pair of stimuli at varying SOAs and required to judge which stimulus was presented first or second^24^,^25^. In this study we evaluated the TBW in obese participants and healthy weight control subjects using both the SJ and TOJ task. We used both tasks as they imply different perceptual and/or cognitive operations. Indeed, while SJ task requires a low-level analysis of stimuli in terms of temporal relationships, TOJ task implies additional processing steps following the lower-level analyses of temporal relationships. Despite such dissimilarity, the two tasks share a common underlying process responsible for the ascription of temporal identity at a stimuli^26^. Such common characteristic allows us to genuinely measure temporal features of multisensory integration in obesity.

## Methods

### Participants

Twenty male obese participants and twenty male healthy-weight participants took part in the study. All participants were right-handed. All obese participants were recruited during the first week of a diagnostic recovery in the IRCCS Istituto Auxologico Italiano–Ospedale San Giuseppe. The healthy-weight group was recruited outside the clinical institute.

The study was approved by the ethical committee of the IRCCS Istituto Auxologico Italiano and it was performed in compliance with Declaration of Helsinki’s ethical principles (World Medical Association, 1991). All participants were volunteers who gave informed written consent, were free to withdraw at will and were naïve to the rationale of the experiment.

Demographic and clinical data are reported in Table 1. The two groups were comparable in terms of *Age* (t(38) = −0.141; p = 0.889; 95% CI [−5.34; 6.14]) and *Education* (t(38) = 0.346; p = 0.731; 95% CI [−1.69; 2.39]). As expected, obese people showed a significant higher *body mass index* (BMI) than healthy participants (t(38) = 12.37; p < 0.001; 95% CI [−22.09; −17.64]).

The Italian version^27^ of the Epworth sleepiness scale was administered to the participants in order to assess subjective average sleep propensity during real-life situations^28^. The two groups reported similar scores at this questionnaire (t(38) = 0.339; p = 0.736; 95% CI [−1.98; 2.78]). Moreover, all participants were assessed by the Italian version^29^ of Beck Depression Inventory^30^ about the presence of depressive symptoms in every-day life. Also in this case, the two groups reported similar scores at this questionnaire t(38) = −0.402; p = 0.69; 95% CI [−3.92; −2.62].

### Stimuli

All stimuli were presented using OpenSesame 2.9.6^31^. Visual stimuli consisted of a white ring circumscribing a visual fixation cross on a black background and were 1.8 cm in diameter or 1.7° of visual angle. They were presented at a distance of approximately 60 cm from the participants and lasted 30 ms. Auditory stimuli consisted of a 3.500 Hz pure tone. They were presented binaurally via noise-cancelling headphones and lasted 30 ms.

### Tasks

**Figure 1:**
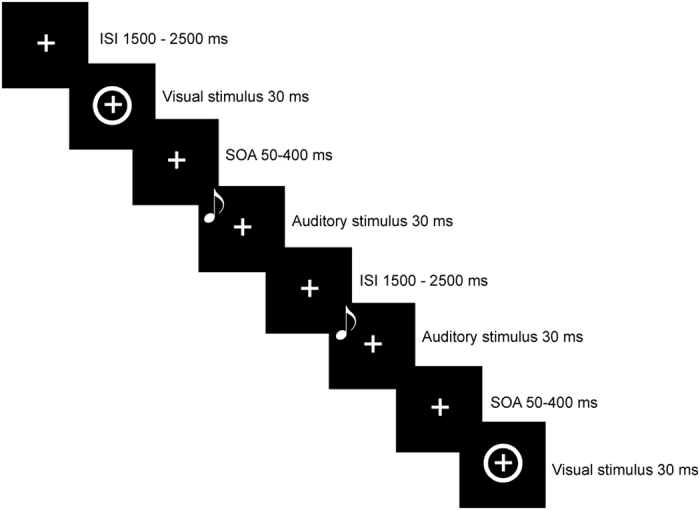
Graphical representation of stimuli presentation.

Participants performed the Simultaneity Judgment Task (SJ) and the Temporal Order Judgment Task (TOJ) in separate sessions. In both tasks (see Fig. 1), visual and auditory stimuli were delivered sequentially with one of the following Stimulus Onset Asynchronies (SOAs): ±50, ±100, ±150, ±200, ±250, ±300, ±350, ±400. Negative SOAs indicate that the auditory stimulus is presented first (auditory leading trials), whereas positive SOAs indicate that the visual stimulus is presented first (visual leading trials). In the SJ task, participants reported whether the auditory and visual stimuli were presented at the same or different times. In the TOJ task, participants reported which stimulus came first. The intertrial interval (ITI) ranged between 2000 and 3000 ms. The presentation of the stimuli was pseudo-randomized.

Participants performed two blocks for each task. In each block, each SOA was presented 16 times for a total of 256 trials per block. Overall participants completed 512 trials for the SJ task and 512 trials for TOJ task. Task order was counterbalanced across participants.

### Procedure

Participants were seated in a dimly lit room with their corporeal midline aligned with a fixation point located 60 cm from the plane of their eyes. Participants rested their right and left index fingers on two response buttons located on a Table. Each hand was in its homonymous hemispace. Participants were instructed to fixate toward a fixation cross at all times. Participants provided their answers by pressing a response button with the right or the left index finger, with the button representation (synchronous/asynchronous or auditory-first/visual-first) being balanced across blocks.

### Data Analysis

To calculate the individual’s TBWs in the SJ task, we first computed the percentage of simultaneous responses across all SOAs for each participant. The observed distribution of responses was fitted to a Gaussian function^32^,^33^ using the *fit* function implemented in MATLAB (fit type: gauss1). The peak of this curve is referred to as the point of subjective simultaneity (PSS). It is assumed that, at this particular SOA, the information from the different modalities is perceived as being maximally simultaneous. Another measure that can be derived from this curve is its standard deviation. The standard deviation is reflected in the width of the curve and is taken as the window of temporal integration (i.e. Temporal Binding Window, TBW), because it represents the range of SOAs at which the brain treats the two sensory information as occurring simultaneously^32^.

For the TOJ task, data analysis was as follows: first we calculated a rate of visual-first responses with each SOA. Then, a single psychometric function was fitted to the response rates across all SOAs, using the *glmfit* function in MATLAB, so as to determine the just noticeable difference (JND) for each group. The JND was defined as half of the difference between the two x values for which the psychometric function had a y value of 25% and 75%^32^.

## Results

### Simultaneity judgment task

**Figure 2:**
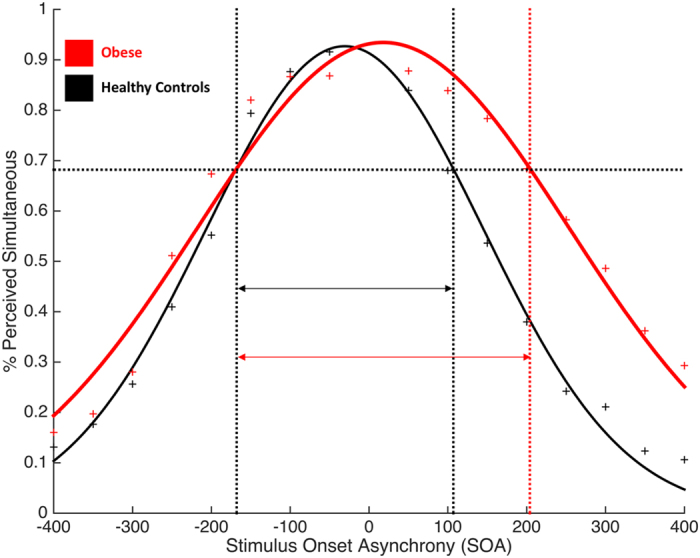
Group mean Temporal Binding Windows defined using the Simultaneity Judgment Task (SJ). Red curve represents obese participants, black curve represents healthy-weight controls. Symbols represent the raw, unfitted data.

Data from one obese participant were excluded from the analysis because of a bad fitting of the Gaussian function, thus making impossible to define his TBW. Data from both groups were normally distributed (Shapiro-Wilk test, p = 0.33), hence parametric statistics were computed. Data were analyzed using an independent sample t-test. The results showed a wider TBW in obese participants (Mean = 663 ms, STD = 128 ms) as compared to healthy-weight controls (Mean = 496 ms, STD = 149; t(37) = −3.73; p < 0.001; 95% CI [−257; −76]) (See Fig. 2).

### Temporal order judgment task

**Figure 3:**
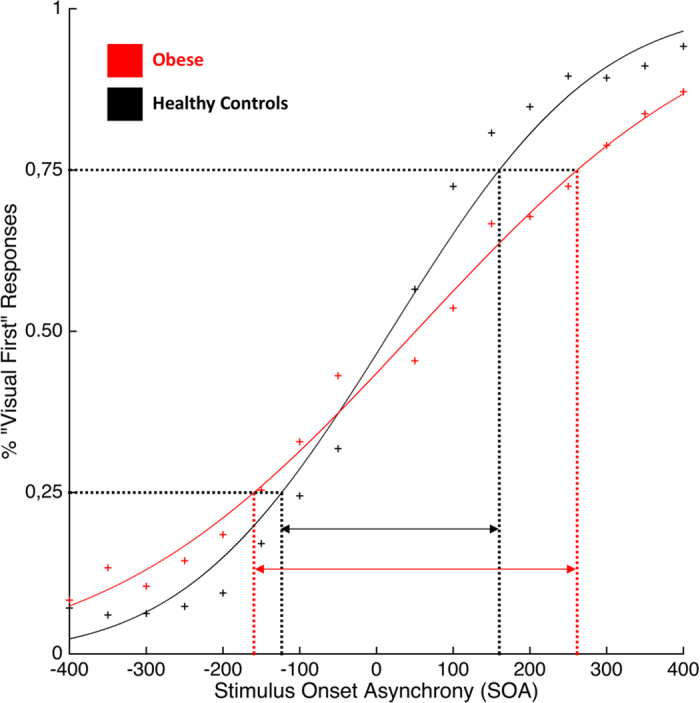
Group mean JND values defined using the Temporal Order Judgment (TOJ) task. Red curve represents obese participants, black curve represents healthy-weight controls. Symbols represent the raw, unfitted data.

JND values violated normality (Shapiro-Wilk test, p = 0.006), hence data were log-transformed to obtain a normal distribution. The JND values were analyzed using an independent sample t-test. The results showed a higher JND in obese participants (Mean = 196 ms; STD = 90 ms) as compared to healthy-weight controls (Mean = 129 ms; STD = 75 ms; t(1, 38) = −3,18; p < 0.003; 95% CI [−0327; −0.07]) (See Fig. 3).

## Discussion

Eating behavior is contributed by almost all our senses. Given the alteration of eating behavior in obesity, we predicted alterations in multisensory integration in obesity. To test our hypothesis we investigated the temporal characteristics of audiovisual integration in obese participants. We found that the Temporal Binding Window (TBW), conceived as a measure of multisensory integration effectiveness^21^, is markedly wider in obese participants as compared to healthy-weight control participants.

Human ability to integrate sensory stimuli from different sources is essential in order to generate a multitude of brain functions^34^ and complex adaptive behaviors^21^, and it affects how we perceive the world^35^. Multisensory integration determines the span-life nature of human cognition^36^ as it promotes heightened attention, perceptual processing and memory in adults as well as in infants^37^. Hence, alteration in this process may cascade into high-level cognitive deficits, well described in obese people^38^,^39^,^40^,^41^,^42^,^43^,^44^,^45^.

Most intriguingly, multisensory integration plays a pivotal role in shaping the way in which we represent and experience our body, namely our body representation^46^,^47^. Several lines of evidence support this idea. For instance, Ionta and colleagues^48^ showed that impaired information from on sensory channel deteriorates multisensory body representation. Preliminary results suggest altered body representation in obesity^45^,^49^,^50^,^51^,^52^,^53^,^54^,^55^. It is entirely possible that these alterations are not only due to top-down influence of psychological components^56^,^57^, but also to bottom-up sensory integration deficits. Preliminary results about disturbances in body representation were reported in other eating disorders such as in anorexia nervosa^58^,^59^, in which an overestimation of body size was observed. Future studies should investigate the possible relationship between eating disorders and body representation.

Possibly one might argue that multisensory deficits in obesity could be traced to altered unisensory perception. Research indeed suggests altered perception of painful^60^,^61^,^62^, vibratory and thermal stimuli^63^, along with altered perception of information from the inner body, including gastric sensorimotor functions^64^ in obese people. However, these findings are controversial. For instance, Deore and colleagues^65^ reported intact auditory perception in a simple detection task in obese participants, while Wan and colleagues^17^ reported equal ability to detect audio or tactile stimuli in obese and healthy-weigh participants. Hence, research on unisensory processing in obesity has generated conflicting results. Hence we are inclined to believe that multisensory deficits in obesity cannot be entirely attributed to unisensory deficits.

How can we account for altered multisensory integration in obese people, as revealed by a wider TBW? The hypothesis we put forward pertains the effect of inflammation on the brain oscillatory activity, which is the neural mechanism enabling multisensory integration. Although our hypothesis is highly speculative, it seems to be supported by two lines of evidence. First, oscillatory activity of the brain, far from being mere noise, represents an instrument that can be used in sensory processing^66^,^67^ and multisensory integration. Second, obesity is characterized by altered neural oscillatory activity^68^. For Instance, Dubbelink and colleagues^68^ recorded neural oscillatory activity in obese participants and normal weight controls during eyes-closed resting-state condition. Results showed altered neural oscillatory activity in delta (0.5–4 Hz) and beta (13–30 Hz) frequency bands in obese as compare to normal weight controls.

Interestingly, high levels of pro-inflammatory cytokines, as often observed in obese people, impact on brain oscillatory activity. Such an effect is likely to reflect alterations of the excitation/inhibition balance, which is contributed by GABAergic interneurons^69^. Empirical studies have indeed shown that pro-inflammatory cytokines increase the protein expression of GABA transporter type 1 and 3, which are the two important subtypes of GATs responsible for the regulation of extracellular GABA levels in the brain. In particular, GAT1 transporter removes GABA from the synaptic cleft^70^, while GAT3 mediates uptake of GABA from the synaptic cleft by surrounding glial cells^71^. Overall, the removal of GABA from the synaptic cleft is likely to decrease the inhibitory effect of GABA, altering neural oscillatory activity^72^.

If our argument is at stake, one may formulate interesting hypotheses on the effect of inflammation on multisensory processing. For instance it could be hypothesized that participants with ongoing inflammation show altered temporal window of integration, as measured with a simultaneity judgment task, and altered perception of multisensory illusions^73^.

We are aware of the speculative nature of our hypothesis, and that a number of factors play a role in the processes here described. For instance, it must be noticed that the role of oscillatory activity in multisensory integration is still a matter of debate. It might be argued that altered stimulus-driven neural response, rather than altered oscillatory activity in obese participants might account for our results. Such an explanation is not at odds with our proposal. Stimulus-driven neural response and oscillatory activity are tightly linked to each other, so that the former depends on the latter at the time the stimulus impinges on brain oscillatory activity^74^.

In conclusion, this study suggests that temporal integration of auditory and visual stimuli is altered in obesity. Many basic questions regarding the relationship between obesity and multisensory integration as possible consequence effect on cognition remain unanswered. Obesity is one of the most significant contemporary health concern characterized by an unhealthy body, such as chronic inflammation, and an unhealthy mind, such as cognitive dysfunctions^75^. Furthermore recognizing possible differences in multisensory abilities in obesity would have several important applications not only in terms of more specific remediation strategies for cognitive difficulties, but also for the future design and implementation of healthy assistance programs and devices based on multisensory perception^76^.

## Additional Information

**How to cite this article**: Scarpina, F., *et al.* Altered multisensory temporal integration in obesity. *Sci. Rep.* **6**, 28382; doi: 10.1038/srep28382 (2016).

## Figures and Tables

**Table 1:** Demographic and clinical data for the obese participants and the healthy-weight participants.

Group	Age in years	Education in years	BMI	BDI Score	Epworth Score
Obese	37.4	12.8	43.5	6.6	6.1
	(8.9)	(3.9)	(4.52)	(4.6)	(4.4)
Healthy	37.8	13.1	23.6	6	6.5
	(9.8)	(2.1)	(1.9)	(5.5)	(2.7)

Means and standard deviations (in brackets) are reported. Age and Education are reported in years; BMI = body mass index express in units of kg/m^2^; BDI = Beck Depression Inventory.
